# Sex Differences in the Impact of Body Composition and Bone Mineral Content on Cardiopulmonary Performance in Elite Youth Water Polo Athletes

**DOI:** 10.3390/sports14020050

**Published:** 2026-02-02

**Authors:** Regina Benko, Mark Zamodics, Mate Babity, Gusztav Schay, Tamas Leel-Ossy, Zsuzsanna Ladanyi, Timea Turschl, Dorottya Balla, Csongor Mesko, Hajnalka Vago, Attila Kovacs, Eva Hosszu, Szilvia Meszaros, Csaba Horvath, Bela Merkely, Orsolya Kiss

**Affiliations:** 1 Heart and Vascular Center, Semmelweis University, Városmajor Street 68, 1122 Budapest, Hungary; regibenko@gmail.com (R.B.); babity.mate@semmelweis.hu (M.B.);; 2 Department of Sports Medicine, Semmelweis University, Városmajor Street 68, 1122 Budapest, Hungary; 3 Department of Biophysics and Radiation Biology, Semmelweis University, Tűzoltó Street 34-37, 1094 Budapest, Hungary; 4 Department of Internal Medicine and Oncology, Semmelweis University, Korányi Sándor Street 2/a, 1083 Budapest, Hungarymeszaros.szilvia@semmelweis.hu (S.M.);; 5 Pediatric Center, Tűzoltó Street Department, Semmelweis University, Tűzoltó Street 7-9, 1094 Budapest, Hungary

**Keywords:** CPET, body composition, bone mineral density, water polo, athlete

## Abstract

Body composition, bone mineral density, and cardiopulmonary exercise testing (CPET) are commonly used to assess aerobic fitness in athletes, but their interrelationships remain unclear. This study compared these parameters by sex and examined their associations in elite athletes. Our study included 145 youth water polo players (age: 15.7 ± 1.6 years; male: 75). Body composition was measured by DEXA, and treadmill CPET was performed using a sport-specific protocol. We analysed the correlations between the following factors by multivariate linear regression: lean body mass (LBM, LBM_index_); body fat mass (BFM); percent body fat (PBF); bone mineral content (BMC); lumbar, femoral, and radial bone mineral density (LBMD, FNBMD, FTBMD, RBMD); exercise time; absolute and relative maximal oxygen uptake (VO_2absmax_, VO_2relmax_); maximal ventilation (VE_max_). Exercise time was found to be negatively correlated with BFM, while VO_2relmax_ was found to be negatively correlated with BFM and PBF. VO_2absmax_ was found to be positively correlated with BFM, LBM, BMC, FNBMD, and RBMD. VE_max_ was found to be positively correlated with LBM and LBM_index_. In males, VO_2absmax_ and VE_max_ were found to be positively correlated with LBMD and FTBMD. Correlations between bone density and CPET proved to be stronger in males. Our results indicate that body composition and bone density parameters influence CPET parameters, and their complex evaluation can support personalized diagnostics and athletes’ health.

## 1. Introduction

In Hungary, water polo is widely practiced and well-supported, with both youth and adult teams achieving success at international levels. Water polo is considered a high-intensity and mixed sport, characterized by intermittent bursts of effort alternating with short recovery periods, requiring players to sustain engagement throughout the match. Success in this sport demands a combination of aerobic and anaerobic endurance, muscular strength, and advanced technical skills, highlighting the complex interaction between physiological capacities and motor performance. Given the physical demands of elite water polo, both body composition and cardiopulmonary fitness play crucial roles in performance optimization and in supporting evidence-based training and athlete management strategies [1,2].

Body composition, a resting determinant of physical fitness, quantifies the body’s main compartments, including fat mass, fat-free mass, and bone mineral content. Monitoring body composition in athletes is essential for optimizing training, nutrition, and recovery, as changes in muscle and fat mass can significantly influence strength, endurance, and performance efficiency. Extreme or poorly managed alterations may lead to sport-related pathologies, underscoring the importance of regular assessments for both athletic performance and long-term health. Body composition can be assessed using various techniques, among which dual-energy X-ray absorptiometry (DEXA) is widely regarded as one of the most accurate and comprehensive methods. Two-dimensional imaging by DEXA uses low- and high-energy X-ray beams and, by applying the three-compartment (3C) model, enables simultaneous and standardized measurements of lean body mass (LBM), body fat mass (BFM), and bone mineral content (BMC), providing detailed information on both total and regional composition [3]. Importantly, in addition to body composition assessment, DEXA is the most often applied tool for the precise measurements of bone mineral density (BMD). Bone mineral density increases most rapidly during childhood and adolescence, and the level achieved during this period is a key predictor of skeletal strength later in life [4]. In contrast, suboptimal BMD in youth is associated with a higher risk of fractures and contributes to the development of osteoporosis in adulthood [1,5]. During growth, several modifiable factors—such as adequate calcium and vitamin D intake, sunlight exposure, and particularly regular physical activity—substantially contribute to BMD variation during growth [6,7]. On the other hand, the skeletal muscle system of young athletes in development is also at increased risk of damage due to significant physical strain [8]. Moreover, marked sex differences are observable at a young age, regarding the development and structure of the musculoskeletal system and body composition [9]. Despite relying on ionizing radiation, DEXA involves only a minimal effective dose, typically 4–5 µSv for a whole-body scan—less than the average daily background radiation—supporting safe assessments in diverse populations, not only athletes [10].

Complementing these resting examinations, maximal intensity cardiopulmonary exercise testing (CPET) provides a comprehensive assessment of physical fitness, allowing the monitoring of training status, the optimization of exercise programs, and the early detection of potential pathophysiological conditions [11]. This non-invasive method evaluates the integrated function of the cardiovascular, respiratory, and musculoskeletal systems under dynamic exercise [10]. By applying standardized exercise protocols and ventilatory gas exchange analysis while continuously recording physiological parameters—such as heart rate (HR), blood pressure, oxygen saturation (SpO_2_), oxygen uptake (VO_2_), carbon dioxide production (VCO_2_), ventilatory efficiency, and blood lactate levels—CPET delivers objective measures of cardiorespiratory fitness and a detailed profile of exercise capacity, cardiovascular and pulmonary function, and both aerobic and anaerobic metabolic thresholds. It is widely recognized as the reference method for investigating unexplained exercise limitations, providing both diagnostic and prognostic insights and supporting the development of individualized training and rehabilitation strategies [12,13].

Although a number of resources for data are available in the independent literature—on the body composition, bone mineral density, and cardiorespiratory fitness parameters of athletes—less is known about their relationships, particularly in youth athletes [14,15]. Previous research has linked body composition to cardiopulmonary performance, but most studies were conducted in non-athletic or even patient populations, frequently focused on older individuals, making it difficult to extend the findings to elite young athletes [16,17]. These gaps highlight the importance of considering structural and functional measures together when evaluating elite youth athletes. Our first examination in this topic has explored these relationships in female youth water polo players, providing valuable initial insights into the correlations of body composition measured by DEXA and CPET variables, though bone mineral density was not assessed and no male athletes were evaluated in that study [18]. Nevertheless, the relationships between body composition, bone mineral density, and cardiorespiratory performance remain poorly understood, particularly in youth athletes. In the present study, we aimed to investigate the correlations between these parameters, with a specific focus on sex-related differences among elite water polo players.

## 2. Materials and Methods

### 2.1. Study Population

Participants were recruited within a cardiology and performance screening program conducted at Semmelweis University [19]. Athletes were eligible if they were active members of the national youth water polo teams and free of acute symptoms at the time of evaluation. Exclusion criteria comprised acute illness, musculoskeletal injury, or any known medical condition that could influence cardiopulmonary, musculoskeletal, or body composition outcomes, and any pathological findings detected during the cardiology screening that could potentially affect study results. During the screening period, 151 athletes were assessed, and 6 were excluded based on these criteria, leaving 145 participants for analysis. Out of these, 75 male athletes (age: 15.4 ± 1.4 years; age range: 13.9–19 years, training: 15.8 ± 4.1 h/week) and 70 female athletes (age: 16.1 ± 1.6 years; age range: 13.8–20 years, training: 16.8 ± 5.3 h/week) were examined from the same youth water polo age groups.

All participants maintained their regular training schedules prior to testing, and assessments were scheduled to occur at least 12 h after the last training session or competition. Owing to the challenges of integrating complex testing procedures into the athletes’ demanding training schedules, the timing of assessments for female participants was not synchronized with their menstrual cycles. Written informed consent was obtained from all participants and, where applicable, from parents or legal guardians. The study protocol was approved by the Medical Research Council of Hungary (No. IV/10282-1/2020/EKU) and conducted in accordance with the Declaration of Helsinki and Good Clinical Practice.

### 2.2. Cardiovascular Assessment

**Figure 1:**
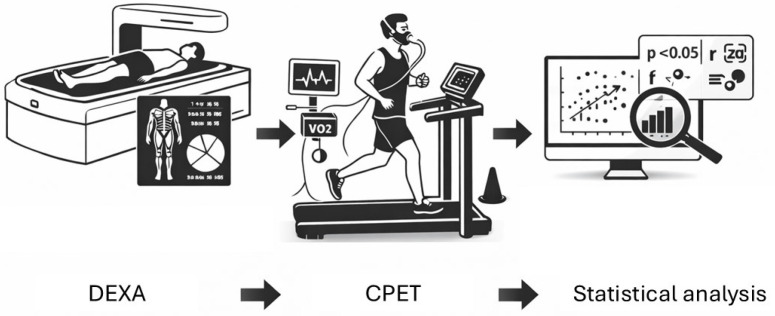
Study overview. Abbreviations: DEXA—dual-energy X-ray absorptiometry; CPET—cardiopulmonary exercise testing.

Athletes participating in the study underwent a comprehensive cardiovascular assessment, beginning with an extensive review of their medical history and a physical examination. Body composition was measured using DEXA (GE-Lunar Corp., Madison, WI, USA), and resting cardiovascular parameters were evaluated via a 12-lead electrocardiogram (ECG) (CardioSoft PC, GE Healthcare, Helsinki, Finland) and blood pressure check (Omron M6 Comfort, OMRON Healthcare Group, Muko City, Japan). Laboratory work, echocardiography (GE Healthcare, Oslo, Norway), and CPET (T-2100, GE Healthcare, Helsinki, Finland) were also performed (Figure 1). All assessments were conducted under the supervision of a cardiology and sports medicine expert.

### 2.3. Body Composition and Bone Mineral Density Analysis

Body composition and bone mineral density were assessed using the Prodigy Dual-Energy X-ray Absorptiometry (DEXA) scanner to determine bone mineral content (BMC), lean body mass (LBM), lean body mass index (LBM_i_), body fat mass (BFM), body fat percentage (PBF), and android-to-gynoid (A/G) fat ratio. Bone mineral density, and the related Z-scores, were measured in the L1-L4 lumbal (LBMD), left femoral neck (FNBMD), left total femoral (FTBMD), and the left radial (RBMD) regions. Participants wore light clothing, and all metal or plastic objects were removed prior to measurement. Standing height was measured to the nearest 0.1 cm using a stadiometer (BSM 370, InBody Co. Ltd., Seoul, Republic of Korea), using the mean of two measurements. Weight was determined with a calibrated digital scale (BSM 370, InBody Co. Ltd., Seoul, Republic of Korea), accurate to 0.1 kg.

### 2.4. Cardiopulmonary Exercise Testing

Athletes completed a standardized treadmill-based running protocol (T-2100, GE Healthcare, Helsinki, Finland) designed to achieve full-intensity exertion. The sport-specific protocol began with a 1 min resting measurement in a seated position, followed by a 2 min warm-up walk at 6 km/h. Subsequently, the treadmill speed was then elevated to 8 km/h and remained constant, while the incline was gradually raised by 1.5% per minute until failure. The protocol ended with a 1 min cool-down walk and a subsequent 4 min seated rest. Testing was ended after 5 min of recovery, following blood pressure and lactate measurements. Respiratory volumes and gas exchange variables, including oxygen uptake (VO_2_) and carbon dioxide production (VCO_2_), were continuously measured (Respiratory Ergostik, Geratherm, Geratal, Germany). A continuous 12-lead ECG (CAM-14 module, GE Healthcare, Finland) was monitored for the whole duration. Capillary blood lactate concentrations were obtained by fingertip sampling at rest, every two minutes during exercise, at maximal workload, and after five minutes of recovery (Lactate Scout 4+, EKF Diagnostik, Barleben, Germany). Blood pressure was measured at rest, every three minutes throughout the exercise protocol, at maximal load, and at one and five minutes of recovery. The following measures were analysed: exercise time, anaerobic threshold time, anaerobic threshold time in percentage of the exercise time, absolute and relative maximum oxygen uptake (VO_2absmax_, VO_2relmax_), relative oxygen uptake at the anaerobic threshold (VO_2relana_), relative oxygen uptake at the anaerobic threshold in percentage of VO_2relmax_ (VO_2relana%_), maximum ventilation (VE_max_), maximum lactate, 5 min recovery lactate, resting heart rate (HR_res_), maximal heart rate (HR_max_), heart rate at the anaerobic threshold (HR_ana_), 1 min recovery heart rate (HR_r1_), 5 min recovery heart rate (HR_r5_), 1 min heart rate recovery (HRR_1_), 1 min heart rate recovery in percentage of the maximal heart rate (HRR_1%_), 5 min heart rate recovery (HRR_5_), 5 min heart rate recovery in percentage of the maximal heart rate (HRR_5%_), resting lactate (Lac_res_), maximum lactate (Lac_max_), and 5 min recovery lactate (Lac_r5_).

### 2.5. Statistical Analysis

R software was used to process the data (R Core Team, 2024., version 4.4., R: A Language and Environment for Statistical Computing. R Foundation for Statistical Computing, Vienna, Austria. https://www.R-project.org/, accessed on 1 April 2024). Multivariate linear regression models were applied to examine the relationships of interest, incorporating the primary predictor variable along with age and height as confounders. The correlation coefficient for the entire dataset was modified to account for the quantity of ordinal and numerical variables included in the models. Correlations were reported as Estimate (Est), indicating the estimated change in response variable for a one-unit change in predictor variable, accompanied by Standard Error (SE). The comparison of baseline anthropometric data between sexes was performed using a two-sample *t*-test. Statistical significance was defined as *p* < 0.05. Descriptive data were presented as mean ± standard deviation (SD).

## 3. Results

### 3.1. Sex Differences in Body Composition, Bone Mineral Density and CPET Parameters

In male athletes, weight, LBM, LBM_i_ and BMC were higher, whereas BFM and PBF were lower compared to females; no sex differences were found in the A/G fat ratio in this young elite athlete population (Table 1). Regarding the bone mineral density measurements, RBMD also proved to be higher in male athletes as compared to females, while no differences were found in LBMD, FNBMD, FTBMD and the related Z-scores (Table 1).

Considering the CPET-derived parameters, the exercise time, anaerobic threshold time, VO_2absmax_, VO_2relmax_, VO_2relana_, VE_max_ and Lac_max_ also proved to be higher, while VO_2relana%_ was lower in male athletes than in females (Table 2). The HR_r1_ proved to be higher, while the HRR_1_ and the HRR_1%_ proved to be lower in female athletes as compared to males. No sex differences were found in anaerobic threshold time in percentage of the exercise time, HR_res_, HR_ana_, HR_max_, HR_r5_, HRR_5_, HRR_5%_, Lac_res_ and Lac_r5_ (Table 2).

### 3.2. Correlations Between Body Composition and CPET Parameters

#### 3.2.1. Exercise Time

We observed a negative correlation between BFM, PBF and exercise duration in both sexes (with height as a negative confounder). Similarly, weight negatively correlated with exercise time in both sexes (with age as a positive confounder in males, and height as a negative confounder in females). In females, a weak negative correlation was found between BMC and exercise time (with height as a negative confounder). In males, LBM and LBM_i_ were negatively correlated with exercise time (with age as a positive confounder in both cases, and height as a negative confounder in the case of LBM_i_) (see data in Supplementary Materials, Table S1).

#### 3.2.2. Maximum Absolute Oxygen Consumption (VO_2absmax_)

**Figure 2:**
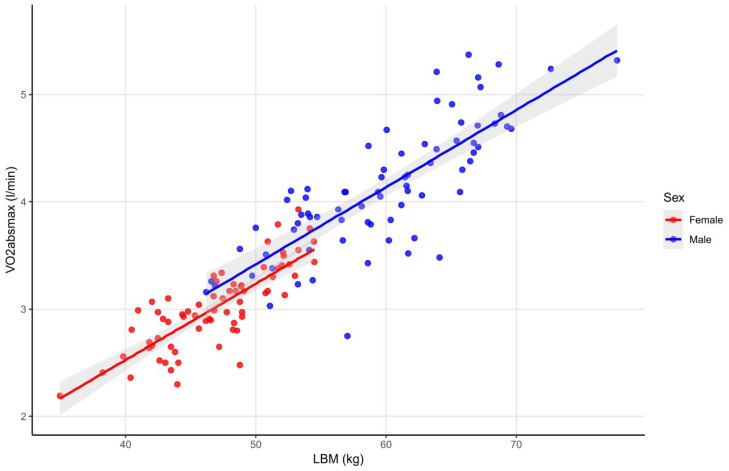
Correlations between LBM and VO_2absmax_ in male and female athletes. The figure illustrates the relationship between lean body mass and maximum absolute oxygen uptake in female and male athletes, showing a strong positive correlation in both sexes, while height proved to be a negative confounder in females. Abbreviations: LBM—lean body mass; VO_2absmax_—absolute maximum oxygen uptake.

The LBM showed a strong positive correlation with VO_2absmax_ in male and in female athletes (with height as a negative confounder in females) (Figure 2). Similarly, LBM_i_ was also positively correlated with VO_2absmax_ in both sexes (with height as a positive confounder in both sexes). Weaker but still positive correlations were observed between BFM (with age as a positive confounder in both sexes and height as a positive confounder in males), BMC, weight and VO_2absmax_. In males, moderate positive correlations were also found between PBF, A/G ratio and VO_2absmax_ (with age and height as positive confounders) (see data in Supplementary Materials, Table S2).

#### 3.2.3. Maximum Relative Oxygen Consumption (VO_2relmax_)

The BFM, PBF and weight were all negatively correlated with VO_2relmax_ in both sexes (neither age nor height showed a significant influence on these relationships). In addition, among females, a weak negative correlation was observed between A/G ratio and VO_2relmax_ (with height as a negative confounder) (see data in Supplementary Materials, Table S3).

#### 3.2.4. Maximal Exercise Ventilation (VE_max_)

**Figure 3:**
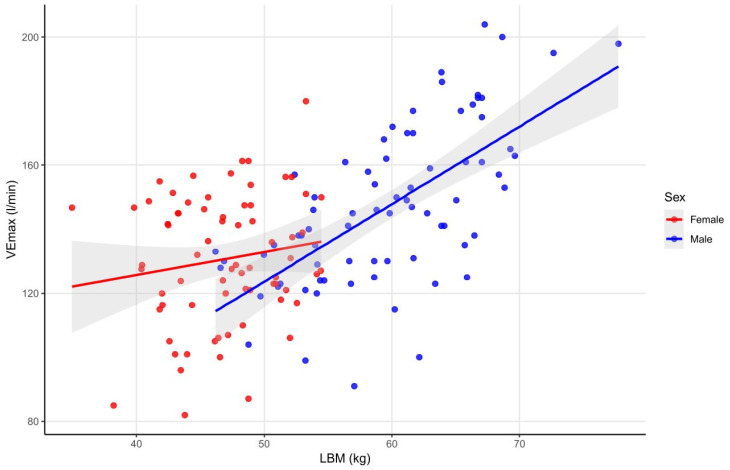
Correlations between LBM and VE_max_ in male and female athletes. The figure illustrates the correlations between lean body mass and maximal ventilation in female and male athletes, demonstrating a positive correlation in both sexes, with a stronger association in males, and with age as a negative confounder in females. Abbreviations: LBM—lean body mass; VE_max_—maximal ventilation.

In both male and in female athletes, LBM positively correlated with VE_max_, with the association being stronger in males (with age as a negative confounder in females) (Figure 3). A similar correlation was observed between LBM_i_ and VE_max_ in both sexes (with height as a positive confounder in males). In males, positive correlations were also found between A/G fat ratio, PBF and VE_max_ (with height as a positive confounder). Weight and BMC also showed positive correlations with VE_max_ in males (this relationship was not influenced by age or height) (see data in Supplementary Materials, Table S4).

### 3.3. Correlations Between Bone Mineral Density and CPET Parameters

#### 3.3.1. Exercise Time

We found a negative correlation between LBMD, LZsc and exercise time in female athletes (with height as a negative confounder). No other correlations were observed between bone mineral density and exercise time (see data in Supplementary Materials, Table S1).

#### 3.3.2. Maximum Absolute Oxygen Consumption (VO_2absmax_)

**Figure 4:**
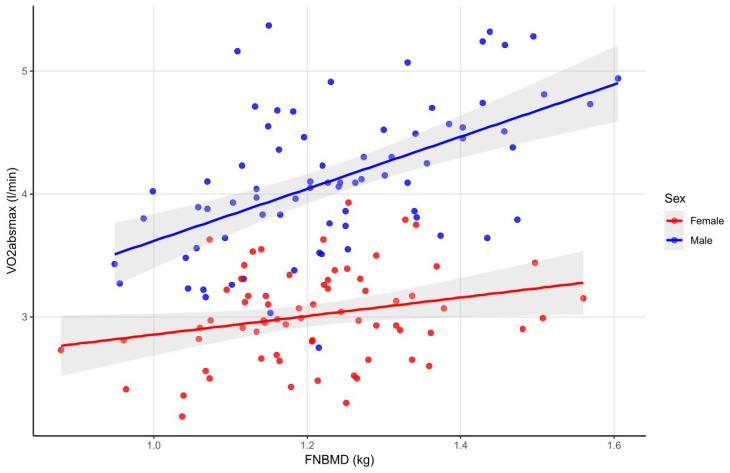
Correlations between FNBMD and VO_2absmax_ in male and female athletes. The figure shows the positive correlations between FNBMD and VO_2absmax_ observed in both sexes, with moderate correlations in males and weaker associations in females, and with height as a positive confounder in males. Abbreviations: FNBMD—femur neck bone mineral density; VO_2absmax_—absolute maximum oxygen uptake.

The FNBMD (Figure 4), FNZsc, FTZsc, and RBMD were positively correlated with VO_2absmax_ in both sexes. In all of these cases, the correlations were weak in females. In males, it consistently appeared as a stronger, but still moderate correlation (with height as a positive confounder in all cases, and age as a positive confounder for FNZsc and FTZsc). In males, a positive correlation was also found between LBMD, FTBMD, and VO_2absmax_, (with age and height both as positive confounders). In females, LZsc showed a weak positive correlation with VO_2absmax_ (with age as a positive confounder) (see data in Supplementary Materials, Table S2).

#### 3.3.3. Maximum Relative Oxygen Consumption (VO_2relmax_)

No correlation was found between bone mineral density data and VO_2relmax_ in either female or male athletes (see data in Supplementary Materials, Table S3).

#### 3.3.4. Maximal Exercise Ventilation (VE_max_)

**Figure 5:**
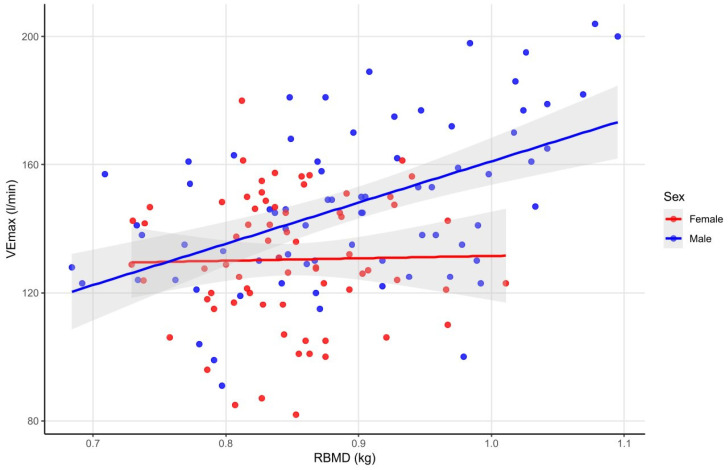
Correlations between RBMD and VE_max_ in male and female athletes. The figure shows no correlation between RBMD and VE_max_ in female athletes, whereas males exhibit a moderate positive correlation, with height as a positive confounder, indicating that higher radial bone mineral density is linked to greater maximal ventilatory capacity. Abbreviations: RBMD—radius bone mineral density; VE_max_—maximal ventilation.

In male athletes, a weak positive correlation was observed between LBMD, FNBMD, FTBMD and VE_max_ (with height as a positive confounder.) A positive and stronger correlation was also found between RBMD and VE_max_ in male athletes (with height as a positive confounder) (Figure 5) (see data in Supplementary Materials, Table S4).

## 4. Discussion

In this study, we examined the impact of body composition and bone mineral density measured by DEXA scanning on cardiopulmonary performance in elite youth water polo athletes, with particular reference to sex differences.

Our findings revealed clear differences in body composition among the examined male and female elite youth water polo athletes. As expected, male athletes demonstrated significantly higher weight, LBM, LBM_i_ and BMC, alongside lower BFM and PBF compared to female athletes. These differences are consistent with normal developmental patterns during adolescence, where hormonal differences lead to greater gains in lean mass in males, whereas females accumulate relatively more fat mass [20,21,22]. No sex differences in the A/G fat ratio were observed, which may reflect that in our highly trained young athletic population the typical sex-specific differences in regional fat distribution are not as pronounced as those described in general untrained adult populations, though no previous study has directly examined this in adolescent sedentary person or athletes [23]. Regarding bone parameters, besides BMC, males showed greater radial bone mineral density, while lumbar and femoral BMD values showed no significant differences. This selective skeletal adaptation may be explained by the sport-specific mechanical loading patterns of water polo, where upper-limb-dominant actions, such as repeated throwing and blocking, together with local muscle strength, may contribute to enhanced radial bone adaptation, particularly in males who generally produce higher absolute forces [24,25,26]. Previous studies in soccer players also highlight the primary role of lean body mass rather than sex in determining bone density. Although these studies are not in water polo specifically, they suggest a mechanism which may partly explain the higher radial BMD observed in male athletes in our study [27].

Previous research in adolescent athletes has shown that lean soft tissue (lean body mass) is a significant mediator of bone mineral density differences across sports with varying mechanical loading, independent of sex. In studies comparing athletes from weight-bearing versus non-weight-bearing sports, higher lean mass has been associated with greater regional BMD, and when lean mass is controlled, sex differences are reduced. These findings suggest that variations in lean body mass rather than sex per se may partly explain the higher radial BMD observed in male athletes in our study.

On the other hand, the aquatic environment reduces axial loading on the lower extremities, potentially minimizing sex-specific differences in lumbar and femoral regions [5,28]. Finally, regarding the CPET parameters, the higher absolute and relative maximum VO_2_, maximal ventilation and exercise time in males reflect well-established physiological differences that could also be observed in our young age athlete population. These findings align with previous findings that males typically exhibit higher maximum VO_2_ due to greater concentration of haemoglobin and higher maximal cardiac output, which increase oxygen transport and utilization [29,30,31]. Regarding VE_max_, it is generally accepted that height and chest size fundamentally determine the amount of air circulated in the lungs during physical exertion [32]. Moreover, in individuals with healthy pulmonary function, ventilatory reserve is generally high, indicating that VE_max_ is not usually the limiting factor of exercise capacity. However, in certain sports, the technique and efficiency of breathing play a crucial role in performance. This is particularly relevant in aquatic disciplines, including water polo, where the alternation between periods spent underwater and above the surface makes the optimization of breathing essential. Males typically exhibit higher VE_max_ than females during maximal intensity exercise, which can be attributed to bigger lung volumes and more powerful respiratory muscles [33,34]. Our results show that this fact is also true for a young-age athlete population like ours. The structural and metabolic differences mentioned above collectively contribute to superior maximal exercise performance in male athletes, resulting in longer exercise duration. Alongside, we also found a higher VO_2relana%_ at the anaerobic threshold in females, which aligns with evidence showing that females rely more on fat oxidation and display lower RER values at submaximal and threshold intensities. This metabolic profile delays the shift toward glycolytic, lactate-producing pathways, allowing the threshold to occur at a higher percentage of their relative maximal oxygen uptake, even if their total VO_2relmax_ remains lower [35]. In the context of the above-mentioned metabolic differences, male athletes also showed higher maximal lactate levels, while resting and recovery values did not differ between sexes. Previous studies in athletes generally reported no significant sex differences in maximal blood lactate; however, these studies often had small sample sizes [36]. This suggests that the higher maximal lactate observed in our male athletes could be due to their greater muscle mass. Lastly, the observed sex differences in HRR_1_, HR_r1_, and HRR_1%_ are likely related to the significantly higher VO_2max_ in male athletes, as HRR_1_ is considered a sensitive indicator of aerobic fitness, whereas other heart rate parameters (HR_res_, HR_max_, HR_ana_, HR_r5_, HRR_5_, HRR_5%_) showed no sex differences [37,38].

In the next step, we examined the correlations between the examined body composition and CPET parameters. The first examined parameter, exercise duration, as the final output of cardiopulmonary fitness during exercise testing, was influenced by several body composition parameters in our study. The BFM and PBF showed the strongest negative effect, likely because it represents non-contractile tissue that adds extra load to the working muscles, increasing the metabolic demand during exercise [39]. A similar mechanism may explain the negative association observed with body weight. The weak negative relationship between BMC and exercise time observed in females may reflect a similar effect: although bone is essential for support and force transmission, the increased bone mass contributes to the load that must be carried during exercise. Nevertheless, this correlation could not be found in male athletes. The negative correlation between LBM and exercise time in males likely reflects pubertal development, as rapid muscle growth during adolescence may outpace cardiovascular adaptations, limiting exercise duration. In contrast, in females, who typically enter puberty earlier and show minor muscle growth as compared to males, increase in LBM was not associated with reduced exercise duration, suggesting that greater muscle mass as a contractile tissue actively working during exercising supports optimal sport-specific adaptations and enhances the capacity to sustain maximal exercise.

The next examined parameter, VO_2absmax_, showed a strong positive correlation with LBM and LBM_i_ in both sexes, reflecting the interconnection of sport-specific adaptations in the muscular and the cardiovascular system. These findings align with research in elite futsal players, where VO_2absmax_ was positively associated with lower-limb lean mass, highlighting the critical role of active muscle tissue in aerobic performance [40]. Weaker, but still positive associations were observed in both male and female athletes between VO_2absmax_ and body weight, BFM and BMC. In males, moderate positive correlations were also found between PBF, A/G ratio and VO_2absmax_. Since fat and bone do not directly contribute to oxygen uptake increase during exercising, this result likely reflects their indirect contribution to the achieved maximal aerobic capacity via total weight. Overall, these findings indicate that resting fitness parameters, particularly LBM, are strong predictors of VO_2absmax_ and could serve as a foundation for establishing reference values in elite athletes. While earlier studies have highlighted the importance of these relationships, comprehensive CPET reference data for this population remain limited [39,41].

In contrast to VO_2absmax_, VO_2relmax_ showed consistent negative associations with fat-related body composition parameters in both sexes. The negative correlations observed with BFM and PBF indicate that higher adiposity is linked to lower relative maximal aerobic capacity. Although VO_2relmax_ was also negatively associated with body weight, the relationship with BFM was stronger, suggesting that the detrimental effect of higher weight on relative aerobic performance is mainly driven by fat mass rather than lean tissue. This is supported by previous observations indicating that excess adiposity impairs relative aerobic performance [16,39]. Additionally, a weak negative association between A/G ratio and VO_2relmax_ was also observed in female athletes. Although comparable patterns have been observed in clinical populations, including individuals with heart failure, this link has not yet been examined in athletes [42]. Overall, these findings emphasize that VO_2relmax_ is primarily influenced by fat-related parameters. Thus, even when LBM and BMC are identical, an increase in BFM may raise VO_2absmax_, but VO_2relmax_ will inevitably decrease due to the greater contribution of fat mass to the total body weight.

Similarly to VO_2absmax_, VE_max_ positively correlated with LBM and LBM_i_ in both sexes, indicating that athletes with greater muscle mass are able to circulate a higher volume of air through their lungs during maximal exertion, confirming the key role of active muscle mass in supporting ventilatory demand during maximal exercise. These findings are in line with previous observations in 10–17 year olds, where VE_max_ increased with FFM, highlighting the role of muscle mass and growth in determining ventilatory capacity during maximal exertion [43]. The stronger association observed in males may reflect sex-related differences in ventilatory capacity, as larger lung volumes and thoracic dimensions may allow maximal ventilation to more closely track metabolically driven ventilatory demand. In males only, additional positive associations with body weight, BMC, PBF, and A/G fat ratio were also observed, likely reflecting that larger body and bone mass, together with greater lean mass, increase the metabolic demand during maximal exercise, which in turn requires higher ventilatory output. In female athletes, where increases in weight are less accompanied by proportional lean mass, these associations were not significant.

Another very important aspect of our investigations was the examination of the correlations between bone density and spiroergometric stress parameters.

First, LBMD and LZsc were negatively correlated with exercise time in females. This finding is consistent with the above-described weak negative relationship between BMC and exercise time observed in females and may be the consequence of the increased bone mass, increasing the load that must be carried during exercise. However, no other correlations were observed between bone mineral density and exercise time.

The present study demonstrated positive correlations between VO_2absmax_ and several site-specific bone parameters in both sexes, with special regard to those bones that take the most active part in sport activity, including FNBMD, FNZsc, FTZsc, and RBMD. Correlations were weak in female athletes; meanwhile, in male athletes, they were stronger and were extended to LBMD and FTBMD, suggesting a more generalized relationship between absolute aerobic capacity and skeletal status. In contrast, VO_2relmax_ showed no significant associations with BMD in either sex. These findings partially align with those reported by El Hage et al. in young adults, who observed clear positive relationships between VO_2absmax_ and multiple bone parameters in both sexes; in that study, VO_2relmax_ correlated with BMC and BMD only in females [44]. They also found that lean mass was a stronger predictor of bone variables than VO_2absmax_ in males, a pattern consistent with our results. The stronger associations observed in our male athletes can likely be explained by higher lean body mass, which is closely linked to VO_2absmax_. Greater lean mass not only increases aerobic capacity but also exerts higher mechanical loading on the skeleton, particularly at weight-bearing and force-transmitting sites. This dual effect likely underlies the stronger VO_2absmax_-BMD correlations in males. Taken together, these findings indicate that absolute aerobic capacity, alongside lean mass, is an important determinant of bone health in adolescent athletes. Promoting VO_2absmax_ and lean mass development during growth may therefore contribute to maximizing peak bone mass and reducing the risk of osteopenia and osteoporosis later in life.

Regarding maximal ventilation, a weak positive correlation was observed between LBMD, FNBMD, FNZsc, FTBMD and VE_max_, and a stronger positive correlation was also found between RBMD and VE_max_ in male athletes. This result is in line with the above-described positive associations between VE_max_ and BMC in male athletes as well as the positive correlations between VE_max_, LBM and LBM_i_ observed in our study. This likely indicates that greater bone mass, along with increased lean mass, elevate metabolic demands during maximal exercise, thereby necessitating higher ventilatory output.

## 5. Conclusions

Our results highlight that body composition and bone density, as resting markers of sport adaptation, are strongly connected to and strongly influence exercise performance. A comprehensive evaluation of these parameters offers valuable insights into an athlete’s physiological condition, supporting the development of personalized diagnostics and training programs, enhancing performance. Incorporating such assessments into routine practice supports both athletes’ health and the individual follow-up of elite athletes. The present study also draws attention to the fact that, when establishing standardized parameters for athletes, it is essential to also take body composition parameters into account. We plan to expand our study by conducting longitudinal assessments to monitor changes in body composition, bone density, and performance over time. In addition, we intend to extend the investigation to other aquatic or mixed sports to better clarify sport-specific adaptation patterns. We also aim to increase the sample size.

### 5.1. Limitations

Our study was carried out on young athletes between the ages of 13 and 20 years who are still developing and have neither reached their peak bone density and mass nor their peak muscle mass. Nevertheless, given the scarcity of scientific evidence on sport-specific adaptations in youth athletes, this aspect adds particular value to our study. The findings cannot be assumed to apply directly to elite athletes in other disciplines, different age categories, or the general population. Despite the limited availability of sport- and age-specific reference data, these findings may provide valuable guidance for assessing athletes involved in similar disciplines.

### 5.2. Geolocation Information

The research was conducted in Budapest, Hungary, at Semmelweis University, with the participation of Hungarian athletes.

## Figures and Tables

**Table 1:** Sex differences in body composition and bone mineral density parameters in elite youth water polo athletes.

	Female	Male	*p*
Weight	68.67 ± 7.86	76.73 ± 9.92	**<0.001**
BFM	19.21 ± 4.52	13.85 ± 4.39	**<0.001**
PBF	27.67 ± 4.13	17.78 ± 4.01	**<0.001**
A/G fat ratio	0.25 ± 0.09	0.26 ± 0.07	0.19
LBM	46.87 ± 4.29	59.81 ± 6.62	**<0.001**
LBM_i_	16.52 ± 1.44	18.59 ± 1.70	**<0.001**
BMC	2.60 ± 0.28	3.08 ± 0.41	**<0.001**
LBMD	1.24 ± 0.12	1.21 ± 0.15	0.12
LZsc	0.83 ± 0.95	0.64 ± 0.96	0.25
FNBMD	1.21 ± 0.13	1.24 ± 0.15	0.22
FNZsc	1.55 ± 0.95	1.63 ± 1.06	0.63
FTBMD	1.15 ± 0.16	1.17 ± 0.13	0.34
FTZsc	0.93 ± 1.03	1.05 ± 0.94	0.48
RBMD	0.85 ± 0.06	0.89 ± 0.10	**<0.001**

Abbreviations: BFM—body fat mass; PBF—percent body fat; A/G fat ratio—android-to-gynoid fat ratio; LBM—lean body mass; LBM_i_—lean body mass index; BMC—bone mineral content; LBMD—lumbal bone mineral density; LZsc—lumbal Z-score; FNBMD—femur neck bone mineral density; FNZsc—femur neck Z-score; FTBMD—femur total bone mineral density; FTZsc—femur total Z-score; RBMD—radius bone mineral density.

**Table 2:** Sex differences in treadmill CPET parameters of elite youth water polo athletes.

	Female	Male	*p*
Exercise time (min)	14.27 ± 3.25	16.94 ± 3.64	**<0.001**
Anaerobic threshold time (min)	9.81 ± 2.66	11.32 ± 3.35	**<0.01**
Anaerobic threshold time %	67.15 ± 17.82	67.28 ± 15.06	0.96
VO_2absmax_ (l/min)	3.02 ± 0.38	4.12 ± 0.60	**<0.001**
VO_2relmax_ (ml/kg/min)	45.21 ± 4.64	54.57 ± 5.07	**<0.001**
VO_2relana_ (ml/kg/min)	40.72 ± 3.69	46.91 ± 5.58	**<0.001**
VO_2relana%_	90.34 ± 5.64	86.00 ± 6.98	**<0.001**
VE_max_ (l/min)	130.61 ± 20.40	147.37 ± 24.92	**<0.001**
HR_res_ (/min)	74.49 ± 10.93	72.73 ± 13.01	0.38
HR_ana_(/min)	183.00 ± 9.01	181.37 ± 10.54	0.32
HR_max_ (/min)	196.06 ± 7.48	197.13 ± 9.27	0.44
HR_r1_ (/min)	169.19 ± 11.99	164.72 ± 14.77	**<0.05**
HRR_1_ (/1 min)	27.07 ± 9.33	32.41 ± 10.28	**<0.01**
HRR_1%_	13.83 ± 4.77	16.51 ± 5.38	**<0.01**
HR_r5_ (/min)	112.19 ± 10.30	113.05 ± 11.32	0.63
HRR_5_ (min)	83.96 ± 8.38	84.08 ± 9.03	0.93
HRR_5%_	42.84 ± 4.35	42.70 ± 4.56	0.85
Lac_res_ (mmol/L)	1.40 ± 0.55	1.31 ± 0.57	0.35
Lac_max_ (mmol/L)	8.29 ± 2.78	9.75 ± 3.22	**<0.01**
Lac_r5_ (mmol/L)	7.33 ± 2.27	7.90 ± 2.75	0.18

Abbreviations: VO_2absmax_—absolute maximum oxygen uptake; VO_2relmax_—relative maximum oxygen uptake; VO_2relana_—relative oxygen uptake at the anaerobic threshold; VO_2relana%_—relative oxygen uptake at the anaerobic threshold in percentage of VO_2relmax_; VE_max_—maximal ventilation; HR_res_—resting heart rate; HR_ana_—heart rate at the anaerobic threshold; HR_max_—maximal heart rate; HR_r1_—1 min recovery heart rate; HRR_1_—1 min heart rate recovery; HRR_1%_—1 min heart rate recovery in percentage of the maximal heart rate; HR_r5_—5 min recovery heart rate; HRR_5_—5 min heart rate recovery; HRR_5%_—5 min heart rate recovery in percentage of the maximal heart rate; Lac_res_—resting lactate; Lac_max_—maximum lactate; Lac_r5_—5 min recovery lactate.

## Data Availability

The datasets generated and analysed in the current study are not available publicly, but are available upon reasonable request from the corresponding author.

## Supplementary Materials

The following supporting information can be downloaded at: https://www.mdpi.com/article/10.3390/sports14020050/s1, Table S1: Correlations between body composition, bone mineral density parameters and exercise time; Table S2: Correlations between body composition, bone mineral density parameters and VO_2absmax_; Table S3: Correlations between body composition, bone mineral density parameters and VO_2relmax_; Table S4: Correlations between body composition, bone mineral density parameters and VE_max_.

## References

[1] Vlachopoulos D., Barker A.R., Ubago-Guisado E., Fatouros I.G., Knapp K.M., Williams C.A., Gracia-Marco L. (2017). Longitudinal Adaptations of Bone Mass, Geometry, and Metabolism in Adolescent Male Athletes: The PRO-BONE Study. J. Bone Miner. Res..

[2] Zamodics M., Babity M., Mihok A., Bognar C., Bucsko-Varga A., Kulcsar P., Boroncsok D., Benko R., Fabian A., Lakatos B. (2025). Evaluation of treadmill cardiopulmonary exercise testing and field measurement results in women’s youth and adult national team water polo players. Heliyon.

[3] Messina C., Albano D., Gitto S., Tofanelli L., Bazzocchi A., Ulivieri F.M., Guglielmi G., Sconfienza L.M. (2020). Body composition with dual energy X-ray absorptiometry: From basics to new tools. Quant. Imaging Med. Surg..

[4] Kalabiska I., Zsakai A., Malina R.M., Szabo T. (2020). Bone Mineral Reference Values for Athletes 11 to 20 Years of Age. Int. J. Environ. Res. Public Health.

[5] Bellver M., Del Rio L., Jovell E., Drobnic F., Trilla A. (2019). Bone mineral density and bone mineral content among female elite athletes. Bone.

[6] Gümüş E., Akgül S., Kanbur N., Derman O. (2019). A comparison of bone mineral density in adolescent swimmers, pentathletes and figure skaters. Turk. J. Pediatr..

[7] Kopiczko A., Adamczyk J.G., Łopuszańska-Dawid M. (2020). Bone Mineral Density in Adolescent Boys: Cross-Sectional Observational Study. Int. J. Environ. Res. Public Health.

[8] Parry G.N., Williams S., McKay C.D., Johnson D.J., Bergeron M.F., Cumming S.P. (2024). Associations between growth, maturation and injury in youth athletes engaged in elite pathways: A scoping review. Br. J. Sports Med..

[9] Gabel L., Macdonald H.M., McKay H.A. (2017). Sex Differences and Growth-Related Adaptations in Bone Microarchitecture, Geometry, Density, and Strength From Childhood to Early Adulthood: A Mixed Longitudinal HR-pQCT Study. J. Bone Miner. Res..

[10] Brazile T.L., Guseh J.S., Baggish A., Hardin C.C., Levine B.D., Shafer K.M. (2025). Cardiopulmonary Exercise Testing. N. Engl. J. Med. Evid..

[11] Dores H., Mendes M., Abreu A., Durazzo A., Rodrigues C., Vilela E., Cunha G., Gomes Pereira J., Bento L., Moreno L. (2024). Cardiopulmonary exercise testing in clinical practice: Principles, applications, and basic interpretation. Rev. Port. Cardiol..

[12] Merkely B.P.A., Tállay A., Vágó H. (2025). Sportorvostan.

[13] Vágó H., Merkely B., Kiss O. (2020). Sportorvostan–Zsebkönyv.

[14] Pettersson S., Kalén A., Gustafsson M., Grau S., Caspers A. (2024). Off- to in-season body composition adaptations in elite male and female endurance and power event athletics competitors: An observational study. BMC Sports Sci. Med. Rehabil..

[15] Squeo M.R., Ferrera A., Monosilio S., Spinelli A., Maestrini V., Mango F., Serdoz A., Zampaglione D., Fiore R., Pelliccia A. (2025). Cardiopulmonary Exercise Testing in Elite Athletes: Rethinking Sports Classification. J. Clin. Med..

[16] Karlsson P., Strand R., Kullberg J., Michaëlsson K., Ahlström H., Lind L., Malinovschi A. (2024). A detailed analysis of body composition in relation to cardiopulmonary exercise test indices. Sci. Rep..

[17] Cooper D.M., Leu S.Y., Taylor-Lucas C., Lu K., Galassetti P., Radom-Aizik S. (2016). Cardiopulmonary Exercise Testing in Children and Adolescents with High Body Mass Index. Pediatr. Exerc. Sci..

[18] Zamodics M., Babity M., Schay G., Leel-Ossy T., Bucsko-Varga A., Kulcsar P., Benko R., Boroncsok D., Fabian A., Ujvari A. (2025). Correlations Between Body Composition and Aerobic Fitness in Elite Female Youth Water Polo Players. Sports.

[19] (2019). Fina Water Polo Rules. https://resources.fina.org/fina/document/2021/02/03/a77f5d7a-0fcd-4dda-88c1-eaa7cf1265ef/final-2020-07-22_fina_wp_manual_2019_-_2021_v24_new.docx.pdf.

[20] Loomba-Albrecht L.A., Styne D.M. (2009). Effect of puberty on body composition. Curr. Opin. Endocrinol. Diabetes Obes..

[21] Wells J.C.K. (2007). Sexual dimorphism of body composition. Best Pract. Res. Clin. Endocrinol. Metab..

[22] Armstrong N., McManus A.M. (2011). Physiology of Elite Young Male Athletes. The Elite Young Athlete.

[23] Bosch T. (2015). Fitness Level is Associated with Sex-Specific Regional Fat Differences in Normal Weight Young Adults. J. Endocrinol. Diabetes.

[24] Tsuji S., Tsunoda N., Yata H., Katsukawa F., Onishi S., Yamazaki H. (1995). Relation between grip strength and radial bone mineral density in young athletes. Arch. Phys. Med. Rehabil..

[25] KutÁČ P., Uchytil J., RygelovÁ M. (2021). The effect of athletic throwing events on the body composition and bone density in the limbs of throwing athletes. J. Sports Med. Phys. Fit..

[26] Kavouras S.A., Magkos F., Yannakoulia M., Perraki M., Karipidou M., Sidossis L.S. (2006). Water polo is associated with an apparent redistribution of bone mass and density from the lower to the upper limbs. Eur. J. Appl. Physiol..

[27] Baker B.S., Chen Z., Larson R.D., Bemben M.G., Bemben D.A. (2020). Sex differences in bone density, geometry, and bone strength of competitive soccer players. J. Musculoskelet. Neuronal Interact..

[28] Gomez-Bruton A., Montero-Marín J., González-Agüero A., Gómez-Cabello A., García-Campayo J., Moreno L.A., Casajús J.A., Vicente-Rodríguez G. (2017). Swimming and peak bone mineral density: A systematic review and meta-analysis. J. Sports Sci..

[29] Zinner C., Sperlich B., Wahl P., Mester J. (2015). Classification of selected cardiopulmonary variables of elite athletes of different age, gender, and disciplines during incremental exercise testing. Springerplus.

[30] Hunter S.K., Angadi S.S., Bhargava A., Harper J., Hirschberg A.L., Levine B.D., Moreau K.L., Nokoff N.J., Stachenfeld N.S., Bermon S. (2023). The Biological Basis of Sex Differences in Athletic Performance: Consensus Statement for the American College of Sports Medicine. Transl. J. Am. Coll. Sports Med..

[31] Skattebo Ø., Martin-Rincon M., Rud B., Nielsen J., Hegg L.H.N., Kleive A.V., Ørtenblad N., Sandbakk Ø., Boushel R., Holmberg H.C. (2025). Determinants of maximal oxygen uptake in highly trained females and males: A mechanistic study of sex differences using advanced invasive methods. J. Physiol..

[32] Prioux J., Ramonatxo M., Mercier J., Granier P., Mercier B., Prefaut C. (2003). Changes in maximal exercise ventilation and breathing pattern in boys during growth: A mixed cross-sectional longitudinal study. Acta Physiol. Scand..

[33] Kilbride E., McLoughlin P., Gallagher C.G., Harty H.R. (2003). Do gender differences exist in the ventilatory response to progressive exercise in males and females of average fitness?. Eur. J. Appl. Physiol..

[34] BenÍTez-MuÑOz J.A., Benito P.J., Cupeiro R., Alcocer-Ayuga M., Rojo-Tirado M.Á., Ramos-Campo D.J., Peinado A.N.A.B. (2025). Differences in Ventilatory Function Based on Cardiorespiratory Fitness and Sex. Med. Sci. Sports Exerc..

[35] Tarnopolsky M.A. (2000). Gender Differences in Substrate Metabolism During Endurance Exercise. Can. J. Appl. Physiol..

[36] Fomin Å., Ahlstrand M., Schill H.G., Lund L.H., Ståhlberg M., Manouras A., Gabrielsen A. (2012). Sex differences in response to maximal exercise stress test in trained adolescents. BMC Pediatr..

[37] de Mendonca G.V., Teodósio C., Bruno P.M. (2017). Sexual dimorphism in heart rate recovery from peak exercise. Eur. J. Appl. Physiol..

[38] Kappus R.M., Ranadive S.M., Yan H., Lane-Cordova A.D., Cook M.D., Sun P., Harvey I.S., Wilund K.R., Woods J.A., Fernhall B. (2015). Sex differences in autonomic function following maximal exercise. Biol. Sex Differ..

[39] Vargas V.Z., Lira C.A.B.d., Vancini R.L., Rayes A.B.R., Andrade M.S. (2018). Fat mass is negatively associated with the physiological ability of tissue to consume oxygen. Mot. Rev. Educ. Fís..

[40] Figueiredo Machado C.L., Nakamura F.Y., de Andrade M.X., dos Santos G.C., Carlet R., Brusco C.M., Reischak-Oliveira A., Voser R.d.C., Pinto R.S. (2023). Total and regional body composition are related with aerobic fitness performance in elite futsal players. J. Bodyw. Mov. Ther..

[41] Sanada K., Kuchiki T., Miyachi M., McGrath K., Higuchi M., Ebashi H. (2006). Effects of age on ventilatory threshold and peak oxygen uptake normalised for regional skeletal muscle mass in Japanese men and women aged 20–80 years. Eur. J. Appl. Physiol..

[42] Dos Santos M.R., da Fonseca G.W.P., Sherveninas L.P., de Souza F.R., Battaglia Filho A.C., Novaes C.E., Pereira R.M.R., Negrão C.E., Barretto A.C.P., Alves M.J.d.N.N. (2020). Android to gynoid fat ratio and its association with functional capacity in male patients with heart failure. ESC Heart Fail..

[43] Armstrong N., Welsman J. (2020). Influence of sex-specific concurrent changes in age, maturity status, and morphological covariates on the development of peak ventilatory variables in 10–17-year-olds. Eur. J. Appl. Physiol..

[44] El Hage R., Zakhem E., Theunynck D., Zunquin G., Bedran F., Sebaaly A., Bachour F., Maalouf G. (2014). Maximal Oxygen Consumption and Bone Mineral Density in a Group of Young Lebanese Adults. J. Clin. Densitom..

