# Supplementary material for: Sex Differences in the Impact of Body Composition and Bone Mineral Content on Cardiopulmonary Performance in Elite Youth Water Polo Athletes
Source: Sports (Basel). 2026 Feb 2;14(2):50. doi: 10.3390/sports14020050 (PMC12944400; doi:10.3390/sports14020050)
Supplement: Supplementary file 1 [file sports-14-00050-s001.zip › Supplement Table S1.pdf]

Correlations between body composition, bone mineral density parameters and exercise time

**Exercise time**	Est	SE	p	Adjusted R^2	Age	Height
Weight (f)	-1.14×10^{-1}	4.90×10^{-2}	<0.05	0.24	no	yes (-)
Weight (m)	-1.38×10^{-1}	4.65×10^{-2}	<0.01	0.14	yes (+)	no
LBM (f)	-9.78×10^{-2}	9.23×10^{-2}	0.29	0.20	no	yes (-)
LBM (m)	-2.08×10^{-1}	7.85×10^{-2}	<0.01	0.12	yes (+)	no
BFM (f)	-2.24×10^{-1}	8.02×10^{-2}	<0.01	0.27	no	yes (-)
BFM (m)	-2.34×10^{-1}	9.22×10^{-2}	<0.05	0.12	no	yes (-)
BMC (f)	-3.18	1.43	<0.05	0.24	no	yes (-)
BMC (m)	-1.26	1.41	0.38	0.05	no	no
A/G fat ratio (f)	-7.26	4.08	0.08	0.22	no	yes (-)
A/G fat ratio (m)	-3.58	6.71	0.59	0.04	no	yes (-)
PBF (f)	-2.34×10^{-1}	8.44×10^{-2}	<0.01	0.27	no	yes (-)
PBF (m)	-2.13×10^{-1}	1.01×10^{-1}	<0.05	0.09	no	yes (-)
LBM_i (f)	-3.46×10^{-1}	2.59×10^{-1}	0.19	0.20	no	yes (-)
LBM_i (m)	-6.74×10^{-1}	2.57×10^{-1}	<0.05	0.12	yes (+)	yes (-)
LBMD (f)	-7.66	2.89	<0.05	0.26	no	yes (-)
LBMD (m)	5.73×10^{-2}	3.13	0.99	0.04	no	yes (-)
LZsc (f)	-9.61×10^{-1}	3.73×10^{-1}	<0.05	0.26	no	yes (-)
LZsc (m)	-2.23×10^{-2}	4.33×10^{-1}	0.96	0.04	no	yes (-)
FNBMD (f)	-3.34	2.77	0.23	0.19	no	yes (-)
FNBMD (m)	-2.11	3.26	0.52	0.04	no	yes (-)
FNZsc (f)	-3.93×10^{-1}	3.91×10^{-1}	0.32	0.20	no	yes (-)
FNZsc (m)	-3.10×10^{-1}	4.05×10^{-1}	0.45	0.05	no	yes (-)
FTBMD (f)	-4.33	2.17	0.05	0.22	no	yes (-)
FTBMD (m)	-3.97	3.46	0.25	0.06	no	yes (-)
FTZsc (f)	-4.52×10^{-1}	3.56×10^{-1}	0.21	0.20	no	yes (-)
FTZsc (m)	-5.64×10^{-1}	4.41×10^{-1}	0.20	0.06	no	yes (-)
RBMD (f)	-1.99	6.56	0.76	0.18	no	yes (-)
RBMD (m)	2.13	5.13	0.68	0.04	no	yes (-)
