# Supplementary material for: Sex Differences in the Impact of Body Composition and Bone Mineral Content on Cardiopulmonary Performance in Elite Youth Water Polo Athletes
Source: Sports (Basel). 2026 Feb 2;14(2):50. doi: 10.3390/sports14020050 (PMC12944400; doi:10.3390/sports14020050)
Supplement: Supplementary file 1 [file sports-14-00050-s001.zip › Supplement Table S2.pdf]

Correlations between body composition, bone mineral density parameters and $VO_{2absmax}$

**$VO_{2absmax}$**	Est	SE	p	Adjusted R^2	Age	Height
Weight (f)	3.69×10^{-2}	4.52×10^{-3}	<0.001	0.52	no	no
Weight (m)	4.18×10^{-2}	5.08×10^{-3}	<0.001	0.62	no	no
LBM (f)	7.69×10^{-2}	6.86×10^{-3}	<0.001	0.67	no	yes (-)
LBM (m)	6.98×10^{-2}	8.47×10^{-3}	<0.001	0.62	no	no
BFM (f)	3.63×10^{-2}	9.68×10^{-3}	<0.001	0.21	yes (+)	no
BFM (m)	5.90×10^{-2}	1.20×10^{-2}	<0.001	0.45	yes (+)	yes (+)
BMC (f)	5.91×10^{-1}	1.72×10^{-1}	<0.001	0.18	no	no
BMC (m)	1.03	1.64×10^{-1}	<0.001	0.52	no	no
A/G fat ratio (f)	1.01	5.10×10^{-1}	0.05	0.09	yes (+)	no
A/G fat ratio (m)	2.67	9.15×10^{-1}	<0.01	0.34	yes (+)	yes (+)
PBF (f)	1.25×10^{-2}	1.11×10^{-2}	0.26	0.06	no	no
PBF (m)	4.52×10^{-2}	1.41×10^{-2}	<0.01	0.35	yes (+)	yes (+)
LBM_i (f)	2.12×10^{-1}	2.01×10^{-2}	<0.001	0.64	no	yes (+)
LBM_i (m)	2.27×10^{-1}	2.80×10^{-2}	<0.001	0.61	no	yes (+)
LBMD (f)	6.08×10^{-1}	3.74×10^{-1}	0.11	0.08	no	no
LBMD (m)	1.08	4.37×10^{-1}	<0.05	0.34	yes (+)	yes (+)
LZsc (f)	1.11×10^{-1}	4.77×10^{-2}	<0.05	0.11	yes (+)	no
LZsc (m)	1.01×10^{-1}	6.18×10^{-2}	0.11	0.31	yes (+)	yes (+)
FNBMD (f)	7.02×10^{-1}	3.41×10^{-1}	<0.05	0.10	no	no
FNBMD (m)	1.51	4.35×10^{-1}	<0.001	0.36	no	yes (+)
FNZsc (f)	1.05×10^{-1}	4.80×10^{-2}	<0.05	0.10	no	no
FNZsc (m)	1.62×10^{-1}	5.53×10^{-2}	<0.01	0.34	yes (+)	yes (+)
FTBMD (f)	4.18×10^{-1}	2.76×10^{-1}	0.13	0.07	no	no
FTBMD (m)	1.36	4.76×10^{-1}	<0.01	0.33	yes (+)	yes (+)
FTZsc (f)	8.84×10^{-2}	4.43×10^{-2}	<0.05	0.09	no	no
FTZsc (m)	1.39×10^{-1}	6.21×10^{-2}	<0.05	0.31	yes (+)	yes (+)
RBMD (f)	1.78	7.93×10^{-1}	<0.05	0.11	no	no
RBMD (m)	2.79	6.61×10^{-1}	<0.001	0.41	no	yes (+)
