# Supplementary material for: Sex Differences in the Impact of Body Composition and Bone Mineral Content on Cardiopulmonary Performance in Elite Youth Water Polo Athletes
Source: Sports (Basel). 2026 Feb 2;14(2):50. doi: 10.3390/sports14020050 (PMC12944400; doi:10.3390/sports14020050)
Supplement: Supplementary file 1 [file sports-14-00050-s001.zip › Supplement Table S3.pdf]

Correlations between body composition, bone mineral density parameters and $VO_{2relmax}$

**$VO_{2relmax}$**	Est	SE	p	Adjusted R^2	Age	Height
Weight (f)	-1.56×10^{-1}	7.65×10^{-2}	<0.05	0.10	no	no
Weight (m)	-1.69×10^{-1}	6.82×10^{-2}	<0.05	0.05	no	no
LBM (f)	6.36×10^{-2}	1.44×10^{-1}	0.66	0.04	no	yes (-)
LBM (m)	-1.66×10^{-1}	1.17×10^{-1}	0.16	-0.001	no	no
BFM (f)	-4.76×10^{-1}	1.18×10^{-1}	<0.001	0.23	no	no
BFM (m)	-4.14×10^{-1}	1.30×10^{-1}	<0.01	0.10	no	no
BMC (f)	-2.90	2.27	0.21	0.06	no	no
BMC (m)	-7.04×10^{-1}	2.04	0.73	-0.03	no	no
A/G fat ratio (f)	-1.58×10^{1}	6.16	<0.05	0.13	no	yes (-)
A/G fat ratio (m)	-1.53×10^{1}	9.52	0.11	0.01	no	no
PBF (f)	-5.86×10^{-1}	1.18×10^{-1}	<0.001	0.30	no	no
PBF (m)	-4.41×10^{-1}	1.41×10^{-1}	<0.01	0.09	no	no
LBM_i (f)	1.01×10^{-1}	4.06×10^{-1}	0.80	0.04	no	yes (-)
LBM_i (m)	-5.38×10^{-1}	3.83×10^{-1}	0.16	-0.002	no	no
LBMD (f)	-8.43	4.58	0.07	0.08	no	yes (-)
LBMD (m)	1.00	4.65	0.83	-0.03	no	no
LZsc (f)	-9.59×10^{-1}	6.02×10^{-1}	0.12	0.09	no	yes (-)
LZsc (m)	3.97×10^{-2}	6.41×10^{-1}	0.95	-0.03	no	no
FNBMD (f)	-2.43	4.32	0.58	0.04	no	yes (-)
FNBMD (m)	3.35	4.69	0.48	-0.02	no	no
FNZsc (f)	-3.78×10^{-1}	6.14×10^{-1}	0.54	0.05	no	yes (-)
FNZsc (m)	3.71×10^{-1}	5.84×10^{-1}	0.53	-0.02	no	no
FTBMD (f)	-3.82	3.43	0.27	0.05	no	yes (-)
FTBMD (m)	-6.15×10^{-1}	5.03	0.90	-0.03	no	no
FTZsc (f)	-6.55×10^{-1}	5.58×10^{-1}	0.24	0.07	no	yes (-)
FTZsc (m)	-1.12×10^{-1}	6.42×10^{-1}	0.86	-0.03	no	no
RBMD (f)	8.06×10^{-1}	1.01×10^{1}	0.94	0.03	no	yes (-)
RBMD (m)	-3.61	7.38	0.63	-0.03	no	no
