# Supplementary material for: Sex Differences in the Impact of Body Composition and Bone Mineral Content on Cardiopulmonary Performance in Elite Youth Water Polo Athletes
Source: Sports (Basel). 2026 Feb 2;14(2):50. doi: 10.3390/sports14020050 (PMC12944400; doi:10.3390/sports14020050)
Supplement: Supplementary file 1 [file sports-14-00050-s001.zip › Supplement Table S4.pdf]

Correlations between body composition, bone mineral density parameters and VE_{max}

**VE_{max}**	Est	SE	p	Adjusted R^2	Age	Height
Weight (f)	2.75×10^{-1}	3.42×10^{-1}	0.42	0.06	yes (-)	no
Weight (m)	1.59	2.60×10^{-1}	<0.001	0.43	no	no
LBM (f)	1.32	6.07×10^{-1}	<0.05	0.12	yes (-)	no
LBM (m)	2.495	4.47×10^{-1}	<0.001	0.39	no	no
BFM (f)	-3.48×10^{-1}	5.71×10^{-1}	0.54	0.06	yes (-)	no
BFM (m)	2.47	5.55×10^{-1}	<0.001	0.32	no	yes (+)
BMC (f)	4.12	1.00×10^{1}	0.68	0.06	yes (-)	no
BMC (m)	3.80×10^{1}	8.07	<0.001	0.33	no	no
A/G fat ratio (f)	-3.20×10^{1}	2.79×10^{1}	0.26	0.07	yes (-)	no
A/G fat ratio (m)	8.79×10^{1}	4.26×10^{1}	<0.05	0.18	no	yes (+)
PBF (f)	-8.83×10^{-1}	5.92×10^{-1}	0.14	0.08	yes (-)	no
PBF (m)	2.04	6.36×10^{-1}	<0.01	0.24	no	yes (+)
LBM_i (f)	3.56	1.72	<0.05	0.11	yes (-)	no
LBM_i (m)	8.05	1.47	<0.001	0.39	no	yes (+)
LBMD (f)	-1.45×10^{1}	2.04×10^{1}	0.48	0.05	yes (-)	no
LBMD (m)	4.69×10^{1}	2.04×10^{1}	<0.05	0.19	no	yes (+)
LZsc (f)	-1.42	2.66	0.59	0.05	yes (-)	no
LZsc (m)	5.65	2.86	0.05	0.18	no	yes (+)
FNBMD (f)	-3.75	1.89×10^{1}	0.84	0.05	yes (-)	no
FNBMD (m)	5.52×10^{1}	2.02×10^{1}	<0.01	0.21	no	yes (+)
FNZsc (f)	-1.21	2.67	0.65	0.05	yes (-)	no
FNZsc (m)	6.37	2.54	<0.05	0.20	no	yes (+)
FTBMD (f)	-1.31	1.51×10^{1}	0.93	0.05	yes (-)	no
FTBMD (m)	4.50×10^{1}	2.21×10^{1}	<0.05	0.18	no	yes (+)
FTZsc (f)	-1.16	2.44	0.64	0.05	yes (-)	no
FTZsc (m)	5.10	2.84	0.08	0.16	no	yes (+)
RBMD (f)	5.48×10^{1}	4.37×10^{1}	0.21	0.07	yes (-)	no
RBMD (m)	1.26×10^{2}	2.99×10^{1}	<0.001	0.30	no	yes (+)
